# Estrogen Promotes Melanogenesis Through Facilitating M2 Macrophage Skewing in Melasma

**DOI:** 10.3390/ijms27136044

**Published:** 2026-07-06

**Authors:** Shen Lin, Linwang Su, Yifei Deng, Yingying Qu, Dongyan Shen, Kun Yao, Qi Wang, Mengting Ouyang, Qingfang Xu

**Affiliations:** Department of Dermato-Venereology, Third Affiliated Hospital of Sun Yat-sen University, Guangzhou 510630, China; linsh23@163.com (S.L.); sulw@mail2.sysu.edu.cn (L.S.); danielgomes61917@gmail.com (Y.D.); quyy6@mail.sysu.edu.cn (Y.Q.); shendy3@mail2.sysu.edu.cn (D.S.); yaok2026@163.com (K.Y.); 15723547117@163.com (Q.W.)

**Keywords:** melasma, estrogen, M2 macrophages polarization, vascular endothelial growth factor, melanogenesis

## Abstract

Although estrogen has been identified to play crucial roles in the development of melasma, the exact mechanism of estrogen’s effect on pigmentation is incompletely elucidated. Recent studies have highlighted the pivotal role of immune cells in melasma. Interestingly, infiltrated macrophages are significantly enhanced in melasma lesions. Estrogen could facilitate M2 polarization. However, whether estrogen could stimulate melanogenesis via skewing M2 phenotype remains unknown. This study attempted to determine the significance and molecular mechanism of estrogen-induced M2 phenotype in melasma. We found that M2 infiltration was significantly increased in melasma lesions compared with perilesional skin. *Arginase 1* was identified as the hub gene, and its expression was positively correlated with that of *microphthalmia-associated transcription factor* and *tyrosinase-related protein 1* in melasma through transcriptome analysis. Moreover, β-estradiol (E2) was confirmed to promote M2 skewing while inhibiting M1 polarization via activating STAT6 signaling. Importantly, E2-induced M2 polarization robustly increased melanogenesis by increasing tyrosinase activity and expression of microphthalmia-associated transcription factor and tyrosinase in melanocytes, which were profoundly inhibited by VEGF knockdown or antagonism both in vitro and in ex vivo skin. Furthermore, VEGF was revealed to enhance melanogenesis through activating p38 MAPK and ERK1/2 signaling pathways in melanocytes. Additionally, dermal VEGF was significantly increased, and most of it colocalized with M2 macrophages in melasma lesions. Crucially, E2 administration potently reversed ovariectomy-decreased M2 skewing and subsequently promoted dermal VEGF expression and epidermal melanogenesis in the mouse tail skin, which were significantly suppressed by macrophage depletion. These findings suggest that estrogen may stimulate melanogenesis in melasma through increasing M2 skewing and VEGF expression and secretion in macrophages.

## 1. Introduction

Melasma is a common acquired hyperpigmentary disorder whose pathogenesis remains poorly elucidated. Among many factors associated with the onset of the disease, female sex hormones, genetic predisposition, and exposure to solar radiation are the most important triggers [1]. The preferential development of melasma during women’s reproductive age and the association of this disease with oral contraceptives and pregnancy suggest that female sex hormones promote the development of melasma [2]. Estrogen and progesterone are two important female sex hormones affecting pigmentation. Most studies have shown that estrogen increases melanin production of human melanocytes cultured in monolayers or 3D constructs, while progesterone decreases it [3]. Therefore, studies about the role of female sex hormones in melasma mainly focus on estrogen. While estrogen had been shown to directly upregulate melanin synthesis in human melanocytes [4], the melanocytes from some donors did not respond to estrogen, even at a high dose [5]. Estrogen was also revealed to indirectly stimulate hyperpigmentation through stimulating neighboring cells around melanocytes to secrete paracrine factors [6]. However, data on skin explants revealed that estrogen could not induce melanogenesis nor melanosome transfer [3]. The exact mechanism of estrogen’s effect on pigmentation is still incompletely elucidated and needs to be investigated. Recently, accumulating evidence suggests that immune disorders and chronic subclinical inflammation play a pivotal role in the pathogenesis of melasma [7]. Melasma lesions are characterized by infiltrates of various immune cells [7]. Histological studies have illustrated that there are increased melanophages in melasma, which are more commonly associated with the mixed type of melasma than the epidermal type [1]. Also, the infiltration of CD68+ macrophages is significantly higher in melasma lesions than in unaffected skin [8], suggesting that macrophages might play a role in melasma. Macrophages are key players in inflammatory diseases through polarization. They are highly plastic and can differentiate into two major polarization states influenced by the surrounding microenvironment: classically activated type 1 macrophages (M1) and alternatively activated type 2 macrophages (M2). M1, which is activated by TH1 cytokines such as lipopolysaccharide (LPS) or interferon- (IFN-) γ, can secrete proinflammatory factors such as tumor necrosis factor-α (TNF-α), interleukin (IL)-1β, and IL-6 [9]. In contrast, IL-4 and IL-13 promote the development of anti-inflammatory M2, producing cytokines including vascular endothelial growth factor (VEGF), transforming growth factor-β (TGF-β), and IL-10 [10]. It has been identified that inflammatory and angiogenic cytokines can affect melanocyte pigmentation [11]. For example, IL-1β decreases MITF expression [12], IL-6 reduces tyrosinase activity [13], and TGF-β1 inhibits melanocyte differentiation and tyrosinase expression [14], whereas VEGF stimulates melanin production in melanocytes [15]. Intriguingly, M2 was revealed to increase melanin synthesis in melanocytes derived from human-induced pluripotent stem cells via secreting VEGF [16]. These data suggest that macrophage polarization might exert an important role in melasma. Importantly, estrogen has been highlighted to promote M2 polarization but inhibit M1 polarization [17]. Thus, it is plausible to hypothesize that estrogen might promote melanogenesis via skewing M2 polarization. To our knowledge, no previous studies have investigated the role of estrogen-induced macrophage polarization in melasma.

In this study, we aimed to investigate the effect of estrogen on macrophage polarization and pigmentation. We found that E2 could stimulate melanogenesis through inducing M2 polarization and VEGF secretion and thereby contribute to melasma.

## 2. Results

### 2.1. M2 Macrophage Infiltration Is Increased and Positively Correlated with Melanogenesis in Melasma

**Figure 1:**
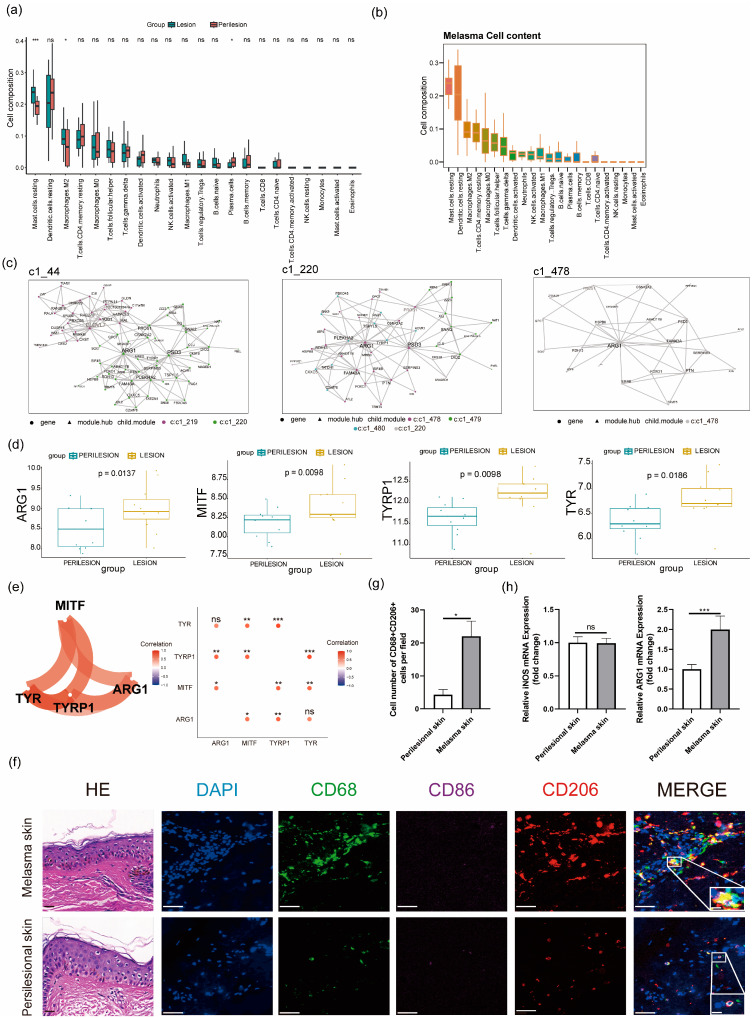
M2 macrophage infiltration and its correlation with hyperpigmentation in melasma lesions. The transcriptome data (GSE72140, n = 11) of melasma lesions vs. perilesional skin were downloaded from the Gene Expression Omnibus (GEO) database. (**a**,**b**) The CIBERSORT R package with the LM22 dataset was used to analyze the infiltration scores of 22 immune cell types between melasma lesions and control (**a**) as well as the proportion of the infiltrated immune cells in melasma lesions (**b**). * *p* < 0.05 and *** *p* < 0.001 by Wilcoxon rank-sum test. ns: no significance. (**c**) Multiscale Embedded Gene Co-expression Network Analysis (MEGCNA) was performed to identify the gene co-expression network and hub genes in melasma among top 5000 genes with the highest average expression levels from the gene expression matrix. (**d**) Paired Wilcoxon rank-sum test was conducted to compare mRNA expression of *ARG1*, *MITF*, *TYRP1*, and *TYR* between melasma skin and perilesional skin. (**e**) The correlation between *ARG1* mRNA level and genes expression of *MITF*, *TYRP1*, and *TYR* in melasma lesions (n = 11). * *p* < 0.05, ** *p* < 0.01, and *** *p* < 0.001 by Pearson correlation coefficient analysis, ns: no significance. (**f**,**g**) Hematoxylin-eosin (HE) examination and multiplex immunofluorescence staining of CD68 (green), CD86 (purple), and CD206 (red) in the perilesional skin and melasma lesions were performed on sections from the same specimen (n = 3). Scale bar = 50 µm. Nuclei were counterstained with DAPI. The white box indicated CD206^+^ CD68^+^ M2 macrophages in skin sections. Scale bar = 10 µm. * *p* < 0.05 by Student’s paired *t*-test. (**h**) RT-qPCR analysis of mRNA expression of *iNOS* and *ARG1* in melasma lesions and perilesional skin (n = 6). *** *p* < 0.001 by Student’s paired *t*-test, ns: no significance.

Sub-clinic inflammation contributes to melasma, and infiltration of immune cells is significantly increased in lesional skin [8]. However, the overall picture of differentiated infiltration of immune cells remains unclear in melasma. We acquired transcriptome data of melasma from the Gene Expression Omnibus database (GEO) (GSE72140), which was analyzed with an estimated immune-cell infiltration analysis of CIBERSORT R. As shown in Figure 1a, only resting mast cells and M2 macrophages were significantly increased, while plasma cells were markedly decreased in lesions compared with those in perilesional skin. However, neither infiltrated M1 nor M0 macrophages were significantly different between lesional and perilesional skin. Moreover, resting mast cells, resting dendritic cells, and M2 macrophages were the top-three largest population of infiltrated immune cells in lesions (Figure 1b), suggesting that M2 phenotype might potentially play a role in melasma. Further, Multiscale Embedded Gene Co-expression Network Analysis (MEGCNA) analysis was performed. Interestingly, *Arginase 1* (*ARG1*) was identified as the hub gene (Figure 1c). The gene expression of *ARG1*, *MITF*, *tyrosinase-related protein 1* (*TYRP1*), and *tyrosinase* (*TYR*) was all significantly upregulated in lesions compared with perilesional skin (Figure 1d). Importantly, *ARG1* expression was positively correlated with that of *MITF* and *TYRP1* (Figure 1e). Considering that ARG1 is the biomarker of M2 phenotype, we suspect that M2 phenotype might be positively correlated with melanogenesis and contribute to melasma.

Further, multiplex immunofluorescence (CD68, CD86, and CD206) was used to study the infiltrated macrophage phenotype in melasma. It was found that immunofluorescence intensity of CD68^+^ cells and CD68^+^ CD206^+^ cells was much stronger in lesional tissues than that in prilesional skin, whereas there was no significant difference in CD68^+^ CD86^+^ cellular fluorescence intensity (Figure 1f). Moreover, *ARG1* mRNA level was confirmed to be obviously increased in lesional skin compared with normal skin (Figure 1g). In contrast, *INOS* gene expression was not significantly changed in melasma skin (Figure 1g). Together, these results demonstrate that M2 macrophage infiltration might be considerably increased in lesions and might contribute to the pigmentation of melasma.

### 2.2. Estrogen Promotes M2 Polarization via STAT6 Signaling in THP-1 Monocyte-Derived Macrophages

**Figure 2:**
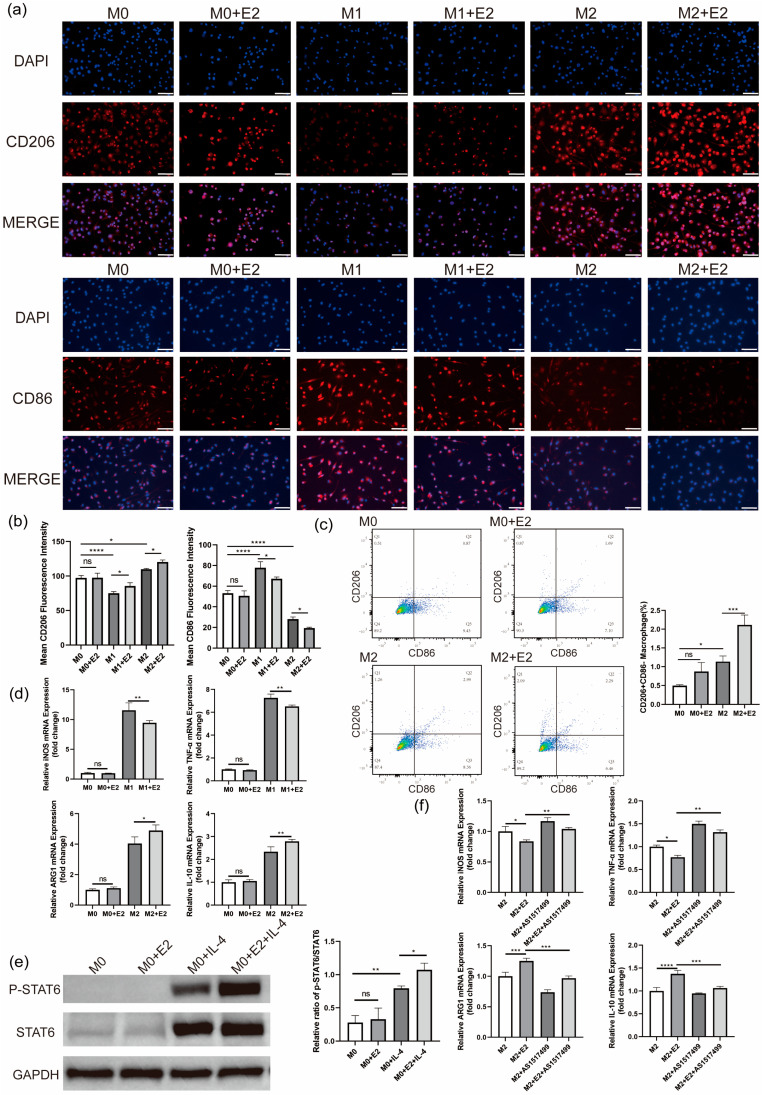
Estrogen stimulates M2 polarization by activating STAT6 pathway in THP-1 monocyte-derived macrophages. (**a**–**d**) Cellular immunofluorescence staining of CD206 and CD86 (Scale bar = 100 µm) (**a**,**b**), flow cytometry analysis of CD206 and CD86 (**c**), and RT-qPCR examination of *iNOS*, *TNF-α*, *ARG1*, and *IL-10* (**d**) in macrophages. THP-1 cells were induced into M0 macrophages after incubation with PMA for 24 h. M0 cells were then pretreated with 10^−7^ M β-estradiol (E2) or left untreated for additional 24 h. Subsequently, cells were polarized into M1 or M2 phenotype and examined or harvested following incubation with 100 ng/mL LPS and 20 ng/mL IFN-γ or 20 ng/mL IL-4 only or in the presence of E2 for 72 h. (**e**) Representative protein expression of phospho-STAT6 (P-STAT6) and STAT6 in M0 macrophages treated with E2 or IL-4 or cotreated with E2 and IL-4. M0 macrophages were differentiated from THP-1 cells according to the method described above. M0 cells were treated with 10^−7^ M E2 or left untreated for 24 h. Then, 20 ng/mL IL-4 was added to cells. Then, 30 min later, cells were collected. GAPDH was used as a protein loading control. (**f**) Representative mRNA expression of *iNOS*, *TNF-α*, *ARG1*, and *IL-10* in M2 macrophages untreated or treated with E2 or cotreated with E2 and STAT6 pathway inhibitor. M0 cells were pretreated with 1μM AS1517499 (STAT6 pathway inhibitor) for 2 h, followed by incubation with or without 10^−7^ M E2 for 24 h. Then, 20 ng/mL IL-4 was added to cells. Next, 72 h later, cells were collected. Data were presented as means ± SD from three independent experiments. Statistical analyses were performed using the one-way ANOVA, followed by a Bonferroni’s multiple comparison test. * *p* < 0.05, ** *p* < 0.01, *** *p* < 0.001, and **** *p* < 0.0001. ns: not significant.

The mechanism of M2 skewing is unclarified in melasma lesions. Estrogen, an important melasma trigger, has been identified to promote M2 polarization [17]. We suspected that estrogen might contribute to M2 phenotype in melasma. To verify the estrogen effect on macrophage phenotype, THP-1 monocyte-derived macrophages were pretreated with 17β-estradiol (E2) and then stimulated with either IFN-γ/lipopolysaccharide (LPS) or IL-4 to induce M1 and M2 polarization, respectively. As expected, CD206 and CD86 fluorescence intensity was dramatically increased by alternative and classical stimulation, respectively (Figure 2a,b). Intriguingly, E2 pretreatment further significantly increased CD206 fluorescence in M2 cells but suppressed CD86 expression in M1 cells. In contrast, E2 incubation did not markedly affect CD206 or CD86 fluorescence in M0 macrophages. Moreover, flow cytometry analysis revealed that E2 preincubation strongly increased the number of CD206^+^ cells in M2+E2 group compared with untreated M2 group (Figure 2c). Additionally, E2 supplement obviously decreased mRNA levels of both *TNF-α* and *INOS*, but increased gene expression of *Arg1* and *IL-10* following stimulation toward either classical or alternative activation (Figure 2d). These results indicate that E2 could promote M2 skewing but inhibit M1 polarization.

How E2 induces M2 skewing remains unknown. IL-4 interacts with the IL-4 receptor and mainly activates signal transducer and activator of transcription 6 (STAT6), leading to macrophage alternative activation [18]. Also, E2-induced M2 polarization was reported to be mediated by STAT6 signaling in bone marrow-derived macrophages of mice [19]. We then studied the significance of STAT6 signaling in E2-induced M2 skewing. As shown in Figure 2e, both STAT6 and phosphorylated STAT6 were markedly increased in E2-treated M2 cells compared with untreated M2 macrophages. However, E2 could not induce STAT6 phosphorylation in M0 macrophages. Crucially, blockade of STAT6 signaling strongly suppressed E2-induced mRNA expression of both Arg1 and IL-10 and reversed E2-inhibited *TNF-α* and *INOS* gene expression in M2 macrophages (Figure 2f). Altogether, these data suggest that E2 could inhibit M1 polarization but promote M2 skewing via STAT6 signaling and might contribute to the increased infiltration of M2 phenotype in melasma lesions.

### 2.3. Estrogen Increases M2-Induced but Decreases M1-Induced Melanogenesis

**Figure 3:**
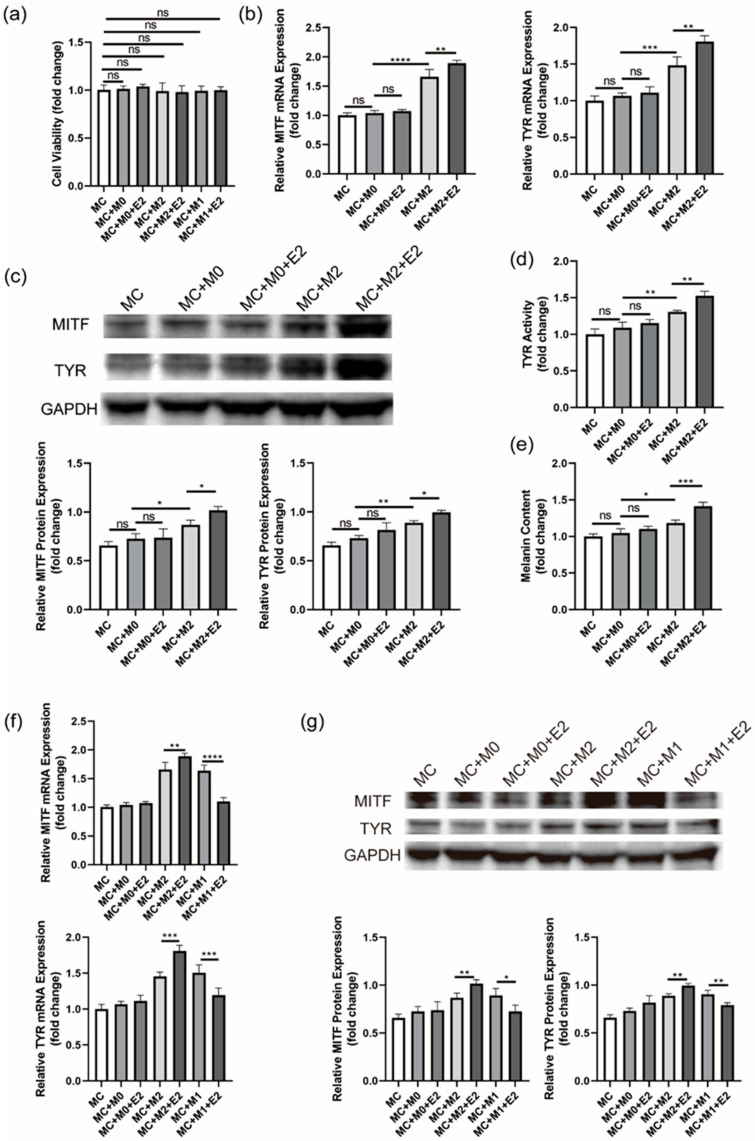
Estrogen promotes M2-induced but inhibits M1-induced melanogenesis. (**a**) The cellular viability of NHEMs treated with the conditional medium (CM) from M0, M1, or M2 phenotype incubated with or without E2. M0, M2, and M1 macrophages were treated with E2 and differentiated from THP-1 cells, as described in Materials and Methods. After polarization, macrophages were washed and incubated with fresh culture medium for 24 h. Then, the conditional medium of macrophages was collected and added to melanocytes (MC) according to the 1:1 ratio of macrophage supernatant and melanocyte culture medium. Melanocytes were incubated with the mixed medium for 48 h and collected. The cellular viability was measured with cell counting kit-8 (CCK-8) assay kit. Also, (**b**) mRNA and (**c**) protein expression of MITF and TYR, (**d**) TYR activity, and (**e**) melanin content were examined in melanocytes treated for 48 h with CM from M0 or M2 macrophages incubated with or without E2. GAPDH was used as a protein loading control. Finally, mRNA (**f**) and protein (**g**) expression of MITF and TYR were measured in melanocytes treated for 48 h with CM from M0, M2, or M1 macrophages treated with or without E2. Data were presented as means ± SD from three independent experiments. * *p* < 0.05, ** *p* < 0.01, *** *p* < 0.001, and **** *p* < 0.0001 by the one-way ANOVA, followed by a Bonferroni’s multiple comparison test. ns: not significant.

After confirming estrogen effect on macrophage phenotype, we then investigated estrogen influence on different phenotype-affected melanogenesis. The supernatant of different macrophage phenotypes was firstly verified not to affect melanocyte viability (Figure 3a). As shown in Figure 3b–e, the supernatant of E2-treated M2 robustly increased melanin content, tyrosinase activity, and mRNA and protein expressions of MITF and tyrosinase compared with other groups, which were not obviously affected by the conditional medium of E2-treated M0 or untreated M0. Although the supernatant of M1 macrophages also obviously elevated both mRNA and protein expressions of MITF and tyrosinase compared with control, these effects were significantly attenuated by E2 treatment (Figure 3f,g). These data suggest that estrogen could enhance M2-induced while inhibit M1-induced melanogenesis and that estrogen might contribute to melasma through stimulating the shift toward M2 phenotype.

### 2.4. Estrogen Promotes Melanogenesis by Stimulating M2 Polarization and VEGF Secretion In Vitro and In Ex Vivo Skin

**Figure 4:**
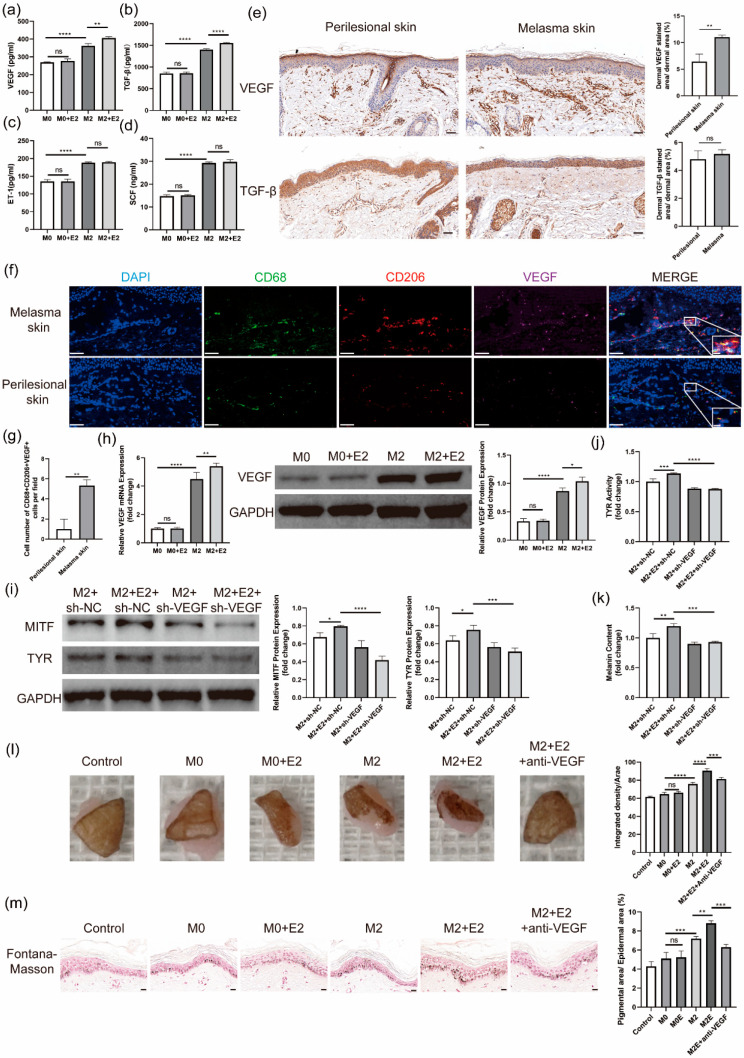
Estrogen increases melanogenesis by stimulating M2 polarization and VEGF secretion. (**a**–**d**) VEGF, TGF-β, stem cell factor (SCF), and endothelin-1 (ET-1) were measured with ELISA in the conditional medium of M0 or M2 macrophages untreated or treated with E2. M0 and M2 macrophages were treated with E2 and differentiated from THP-1 cells, and the conditional medium was collected as described in Materials and Methods. Data were presented as means ± SD from three independent experiments. ** *p* < 0.01 or **** *p* < 0.0001 by the one-way ANOVA, followed by a Bonferroni’s multiple comparison test. ns: not significant. (**e**) Immunohistochemical staining of VEGF and TGF-β in the perilesional skin and melasma lesions and semiquantitative analysis of their expression in the dermis (n = 6). Scale bar = 50 µm. Data were presented as means ± SD. ** *p* < 0.01 by Student’s *t*-test. ns: not significant. (**f**,**g**) Multiplex immunofluorescence staining of CD68 (green), CD206 (red), and VEGF (purple) in the perilesional skin and melasma lesions (n = 3). Nuclei were counterstained with DAPI. Scale bar = 50 µm. The white box indicated CD206^+^ VEGF^+^ CD68^+^ M2 macrophages in skin sections. Scale bar = 10 µm. ** *p* < 0.01 by Student’s paired *t*-test. (**h**) Representative RT-qPCR and Western blotting assay of VEGF in THP-1-derived M0 and M2 macrophages. M0 and M2 macrophages were treated with E2 and differentiated in the same way as in (**a**). Then, cells were harvested from each group for analysis. (**i**–**k**) Protein expression of MITF and TYR (**i**), tyrosinase activity (**j**), and melanin content (**k**) in NHEMs incubated for 48 h with the conditional medium from M2 macrophages differentiated from THP-1 transduced with VEGF-knockdown lentiviral vectors (sh-VEGF) or empty vectors (sh-NC). THP-1 cells were infected with lentiviral vectors at 40 MOI for 12 h. After puromycin selection, stably transfected cells were induced into M0 macrophages by incubation with PMA for 24 h, which were then treated with E2 and differentiated into M2 phenotype as described in Materials and Methods. Afterward, melanocytes were incubated with the conditional medium of M2 cells for 48 h in the same way as described in in Materials and Methods. (**l**,**m**) The conditional medium from E2-treatd M0 or M2 macrophages was untreated or pretreated with 2 μg/mL anti-VEGF165/VEGF121 antibody for 30 min. After preparation, ex vivo human skin was cultured with the medium containing 50% CM from macrophages for 5 days and harvested. Skin samples were photographed with a digital camera after 5-day culture. The skin pigmentation in photos was quantified by integrated density per area using ImageJ software (National Institutes of Health, Bethesda, MD, USA) (**l**). Melanin content was examined with Fontana-Masson staining in each group (**m**). Scale bar = 50 µm. Data were presented as means ± SD from three independent experiments. * *p* < 0.05, ** *p* < 0.01, *** *p* < 0.001, or **** *p* < 0.0001 by the one-way ANOVA, followed by a Bonferroni’s multiple comparison test. ns: not significant.

Estrogen has long been considered to play a crucial role in melasma, yet the molecular mechanism is unclear. We then studied the underlying molecular mechanism of enhanced melanogenesis by E2-induced M2 macrophages. M2 macrophages produce higher levels of angiogenic growth factors and pro-fibrotic cytokines than M1 macrophages to promote tissue remodeling and healing, especially VEGF and TGF-β [20,21], which can also affect melanin synthesis. Although IL-10 expression is increased in M2 macrophages, it has not been found to directly affect melanogenesis. Additionally, stem cell factor (SCF) and endothelin-1 (ET-1) are expressed in macrophages [22,23], which have been highlighted to exert important roles in melasma [24]. Thus, we firstly examined the E2 effect on the secretion of VEGF, TGF-β, SCF, and ET-1 in M2 macrophages. As shown in Figure 4a,b, levels of both VEGF and TGF-β were significantly increased in the conditioned medium from E2-treated M2 cells than those from untreated M2. Although both SCF and ET-1 levels were markedly increased in supernatants of E2-treated or untreated M2 macrophages compared with M0 preincubated with or without E2, no significant difference in their content was observed between E2-treated and untreated M2 groups (Figure 4c,d). These results suggest that E2 could only stimulate M2 macrophages to secrete VEGF and TGF-β. Further, both immunohistochemistry and immunofluorescence staining showed that VEGF was mainly localized in the dermis, and most of it colocalized with CD68^+^ CD206^+^ cells in melasma lesions (Figure 4e,f). Both dermal VEGF expression and CD68^+^ CD206^+^ VEGF^+^ cells were significantly increased in melasma lesions than those in perilesional skin, whereas no difference in TGF-β level was observed, indicating that VEGF but not TGF-β might link with pigmentation and that upregulated VEGF might be related to M2 macrophages in melasma. Thus, we focused on VEGF in our next studies. Similarly, VEGF mRNA and protein expression were also found to be significantly increased in E2-treated M2 macrophages compared with other groups (Figure 4g). To verify the specific role of VEGF in melanogenesis, we knocked down VEGF in THP-1 cells. VEGF mRNA and protein expression were corroborated to be efficiently downregulated in sh-lentivirus-transduced THP-1 cells (Supplementary Figure S1a,b). Importantly, VEGF knockdown profoundly attenuated estrogen-induced expression of MITF and tyrosinase, tyrosinase activity, and melanin content in melanocytes (Figure 4h–j).

To further verify the pro-melanogenic effect of E2-treated M2 macrophages via producing and secreting VEGF, ex vivo human skin was treated with their conditioned medium and anti-VEGF. Although there are five members in the VEGF family—VEGF-A, -B, -C, -D, and placenta growth factor—most attention is focused on VEGF-A due to its dominant role in regulating angiogenesis during homeostasis and disease [25]. Since VEGF165 and VEGF121 are soluble-secreted forms of VEGF-A [26], we used the antibody against VEGF165 and VEGF121 to study the VEGF effect on pigmentation. We showed that both skin pigmentation and epidermal melanin level were not significantly altered by the conditioned medium from either M0 or E2-treated M0 but robustly enhanced by that from both untreated M2 and E2-treated M2 compared with control (Figure 4k,l). Consisting with in vitro results, the conditioned medium of E2-treated M2 significantly increased skin pigmentation and epidermal melanin content compared with untreated M2, which was profoundly abrogated by anti-VEGF. Collectively, these data strongly indicate that estrogen could promote melanogenesis through stimulating M2 polarization and VEGF secretion.

### 2.5. VEGF Regulates Estrogen-Induced Melanogenesis by Binding to Its Receptor and Activating MAPK Signaling in Melanocytes

**Figure 5:**
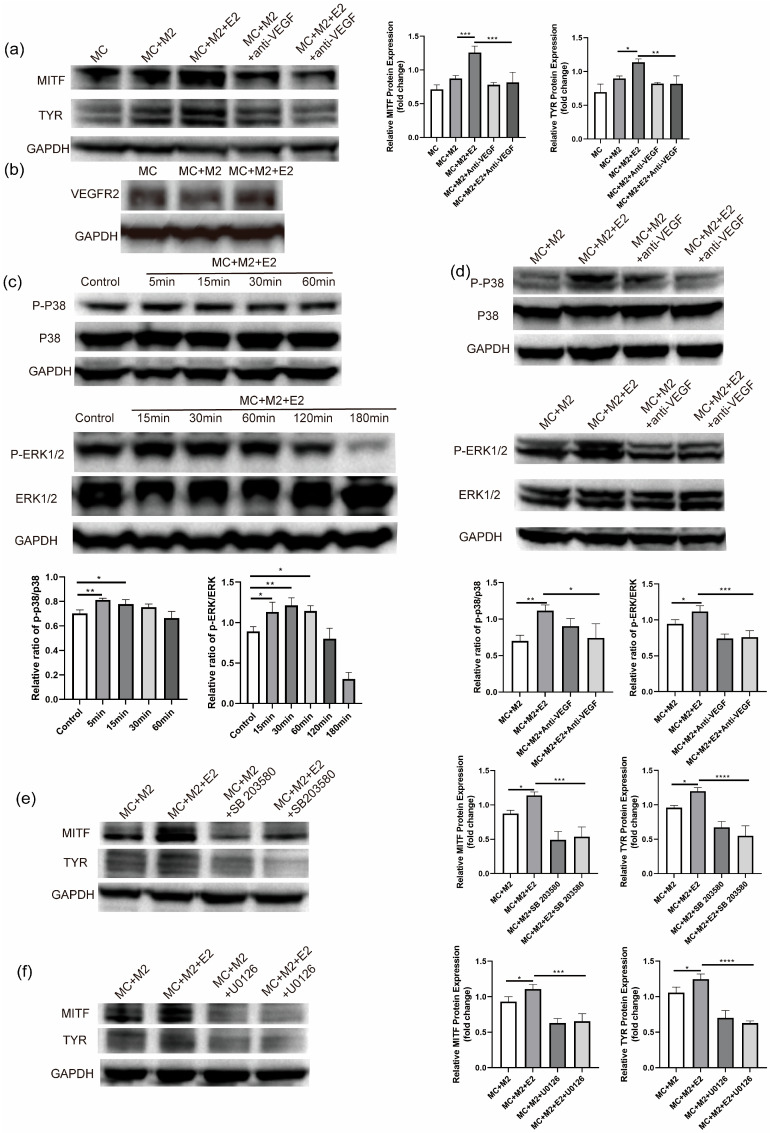
VEGF promotes melanogenesis via binding to its receptor and activating MAPK signaling in melanocytes. (**a**) Representative Western blotting assay of MITF and TYR in NHEMs cotreated with the conditional medium (CM) of M2 or E2-induced M2 and the antibody against VEGF165/VEGF121. CM was collected from M2 or E2-induced M2 according to the method described in Materials and Methods. Following this, CM was pretreated with 2 μg/mL anti-VEGF165/VEGF121 antibody for 30 min and then used to treat melanocytes for 48 h in a 1:1 ratio of melanocyte culture medium. The protein expression of the receptor 2 of VEGF was also examined in melanocytes untreated or treated with CM from M2 or E2-induced M2 for 48 h (**b**). (**c**) Melanocytes were untreated or treated with CM from E2-induced M2 and harvested at the indicated times. Phospho-p38 MAPK (p-p38) and phospho-ERK1/2 (p-ERK1/2) levels were measured with Western blotting. (**d**) Melanocytes were cotreated with CM of M2 or E2-induced M2 and anti-VEGF165/VEGF121 antibody in the same way as in (**a**). Then, cellular lysates were collected at 5 min and 30 min after the treatment for Western blotting analysis of p-p38 and p-ERK1/2, respectively. (**e**,**f**) Protein expression of MITF and TYR in melanocytes cotreated with CM of M2 or E2-induced M2 and inhibitors of MAPK signaling. Melanocytes were preincubated with 30 μM SB203580 (**e**) or 10μM U0126 (**f**) for 2 h and then were incubated with CM of M2 or E2-induced M2 in the presence of SB203580 or U0126 for another 48 h. GAPDH was used as a protein loading control. Data were presented as means ± SD from three independent experiments. * *p* < 0.05, ** *p* < 0.01, *** *p* < 0.001, or **** *p* < 0.0001 by the one-way ANOVA, followed by a Bonferroni’s multiple comparison test.

To further elucidate the molecular mechanism responsible for estrogen-induced melanogenesis, the effect of VEGF on pigmentation and related signaling pathways was examined in NHEMs. We found that VEGF antagonism strongly abrogated the increased protein expression of MITF and TYR induced by E2-skewed M2 macrophages (Figure 5a), indicating that VEGF is pivotal in estrogen-induced melanogenesis. VEGF-A binds to VEGF receptor-1/2 (VEGFR1/2) and subsequently activates phosphoinositide 3-kinase (PI3K)/Akt and p38 and ERK1/2 MAPKs [27]. Interestingly, NHEMs express VEGFR-2 [15]. Importantly, MAPKs are crucial in melanogenesis [28]. Thus, we examined the potential involvement of MAPKs signaling in melanogenesis induced by E2-skewed M2 macrophages. As shown in Figure 5b, VEGFR-2 protein expression was confirmed in NHEMs. Intriguingly, phosphorylation of p38 MAPK markedly increased between 5 and 15 min, whereas phosphorylated ERK 1/2 upregulated between 15 and 60 min in melanocytes following incubation of conditioned medium from E2-treated M2 phenotype (Figure 5c), which were significantly suppressed by VEGF antagonism (Figure 5d). Notably, inhibition of either p38 MAPK or ERK1/2 signaling substantially ameliorated the protein expression of MITF and TYR induced by E2-incubated M2 macrophages (Figure 5e,f). Taken together, these results suggest that VEGF/VEGF receptors/MAPKs axis may play an important role in the pro-melanogenesis by E2-treated M2 macrophages.

### 2.6. Estrogen May Stimulate Melanogenesis via Inducing M2 Phenotype and Inhibiting M1 Transition in Mice

**Figure 6:**
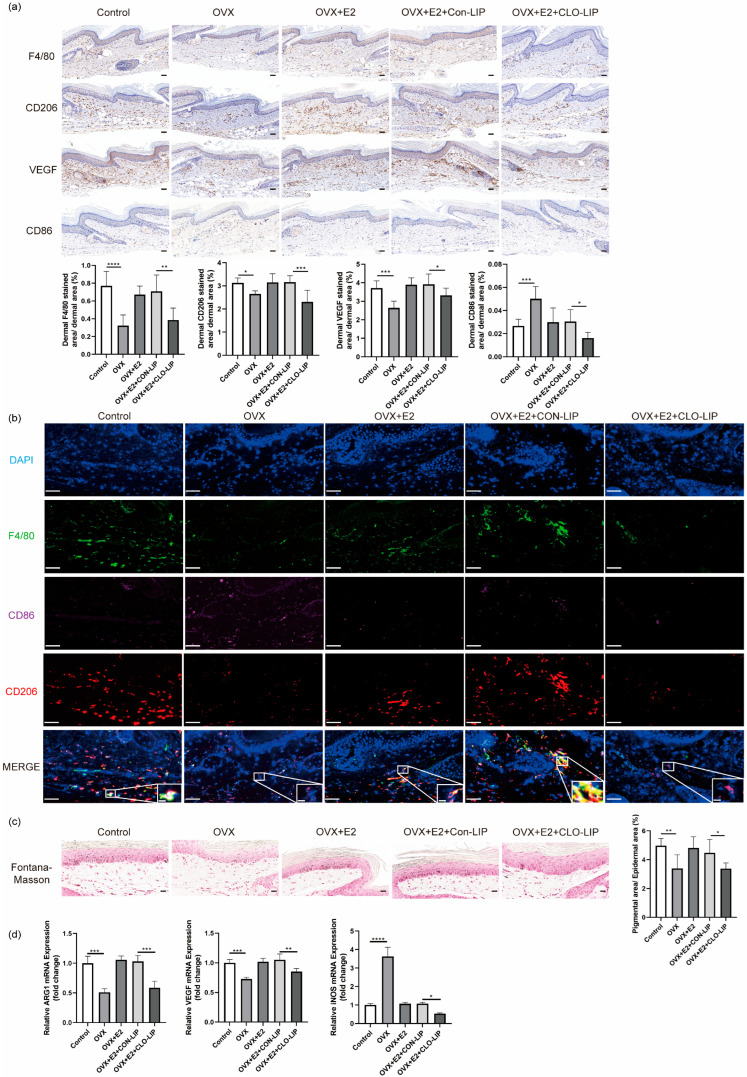
Estrogen promotes melanogenesis via inducing M2 phenotype and VEGF expression in mice skin. Female C57BL/6J mice aged 8 weeks were used. Mice were randomly divided into five groups: Control (received sham surgery), OVX (received ovariectomy), OVX + E2 (received ovariectomy and E2 pellet), OVX + E2 + Con-lip (received ovariectomy, E2 pellet, and control liposome), and OVX + E2 + Clo-lip (received ovariectomy, E2 pellet, and clodronate liposomes). Each group had six mice. Ovariectomy or sham surgery was performed under anesthesia with 2′,2′,2′-tribromoethanol (250 mg/kg). Meanwhile, slow-release E2 pellets were implanted subcutaneously in the back area (0.18 mg, 60-day release pellets) immediately after ovariectomy. Then, 3 days later, control or clodronate liposomes were administered intraperitoneally twice a week for 6 weeks, with the starting dose of 200 μL followed by 150 μL for each subsequent injection. Mice were then killed, and tail skin was harvested. (**a**) Representative immunohistochemical analysis of F4/80, CD206, VEGF, and CD86 in the tail skin of each group. Scale bar = 50 µm. (**b**) Multiplex immunofluorescence staining of F4/80 (green), CD86 (purple), and CD206 (red) in the tail skin. Scale bar = 50 µm. Nuclei were counterstained with DAPI. The white box indicated CD206^+^ F4/80^+^ M2 macrophages in the skin sections. Scale bar = 10 µm. (**c**) Melanin content was evaluated with Fontana-Masson staining in the tail skin. Scale bar = 50 µm. (**d**) RT-qPCR analysis of mRNA expression of *ARG1*, *VEGF*, and *iNOS* in the tail skin. Data were presented as means ± SD from six mice. Statistical analyses were performed with the one-way ANOVA, followed by a Bonferroni’s multiple comparison test, * *p* < 0.05, ** *p* < 0.01, *** *p* < 0.001, and **** *p* < 0.0001.

To better characterize estrogen’s effect on macrophage phenotype and melanogenesis, C57BL/6J mice were ovariectomized and macrophage-depleted. Both dermal CD206 and VEGF levels were sharply reduced, while dermal CD86 expression was significantly enhanced in the tail skin of ovariectomized mice compared with sham control (Figure 6a). Notably, E2 administration in ovariectomized mice effectively reversed these alterations. Moreover, immunofluorescence assay showed that dermal CD206^+^ F4/80^+^ cell number decreased, but CD86^+^ F4/80^+^ cell number increased in ovariectomized mice compared with sham control, which were rescued by E2 supplement (Figure 6b). Meanwhile, epidermal pigmentation was substantially ameliorated in ovariectomized mice compared with sham mice, which, however, was resumed by E2 addition (Figure 6c). These results indicate that estrogen may promote M2 skewing, dermal VEGF expression, and epidermal melanogenesis, while inhibiting M1 polarization in the mouse skin. To identify the macrophage role in E2-induced dermal VEGF expression and epidermal melanogenesis, we depleted mouse macrophages. Clodronate-containing liposomes significantly decreased dermal F4/80, CD206, and CD86 expression in mice cotreated with ovariectomy and E2 compared with control liposomes (Figure 6a,c), indicating the efficient macrophage depletion. Additionally, E2 addition significantly increased mRNA expression of *ARG1* and *VEGF* but decreased *iNOS* mRNA level in the tail skin of ovariectomized mice, which were all obviously inhibited by the macrophage depletion (Figure 6d). Particularly, dermal VEGF expression and epidermal melanin level were markedly reduced by macrophage depletion in mice cotreated with ovariectomy and E2 (Figure 6a,c), implying the important role of macrophages in E2-induced VEGF expression and melanogenesis. Collectively, these results further confirmed that estrogen may stimulate melanogenesis, probably through facilitating M2 skewing and VEGF expression.

## 3. Discussion

Estrogen has been identified to promote the development of melasma through stimulating melanogenesis. However, the exact mechanism of estrogen-induced pigmentation has yet to be fully clarified. Recent studies have highlighted the pivotal role of immune cells in melasma [7]. Intriguingly, infiltrated macrophages are significantly enhanced in melasma lesions [7]. Notably, estrogen can facilitate M2 polarization [17]. The present study found that estrogen might stimulate melanogenesis and contribute to melasma, probably through promoting M2 skewing and VEGF expression.

Although both the infiltrated macrophages and melanophages are increased in melasma lesions, their phenotype remains unclear. We observed that M2 macrophages, but not M1 phenotype, were significantly increased in melasma lesions and that *ARG1* was identified as the hub gene. Moreover, *ARG1* expression was positively correlated with that of *MITF* and *TYRP1.* These findings suggest that increased infiltration of macrophages might be mainly M2 phenotype and link with the pigmentation of melasma. The trigger of M2 skewing is unknown in melasma. Photoaging is considered as one of the main causes of melasma [7]. However, the M1-to-M2 ratio was significantly increased in photoaged skin [29]. Although estrogen was shown to inhibit M2 polarization in a tumor-associated macrophage cell line [30], most studies have identified that estrogen could promote M2 skewing [31]. For example, estrogen could induce alternative macrophage activation in murine bone marrow-derived macrophages and THP-1 macrophages through binding to estrogen receptor-α [17]; E2 regulated macrophages toward a M2 phenotype through superoxide signaling in the myocardial infarction area of mice [32]; E2 upregulated M2 polarization of mouse bone marrow-derived macrophages via enhancing heme oxygenase-1 expression and STAT6 signaling [19]. Thus, we speculated that estrogen might be responsible for M2 polarization in melasma lesions. Indeed, our results confirmed that E2 significantly increased the expression of M2 markers but decreased the levels of M1 markers in vitro, respectively. Moreover, STAT6 signaling was shown to mediate E2-induced M2 polarization. Further, both M2 number and the expression of M2 markers were evidently decreased following ovariectomy, which were potently reversed by E2 supplement in the mouse tail skin. Interestingly, E2 administration obviously reduced ovariectomy-increased M1 skewing. These data verify that E2 could promote M2 polarization but inhibit M1 phenotype both in vitro and in vivo.

Macrophages are highly plastic and exhibit an important role in a wide variety of physiologic and pathologic processes via polarization and subsequently secreting cytokines [9]. Strikingly, the melanogenesis is largely affected by inflammatory and angiogenic cytokines [11]. We then investigated the effect of estrogen-induced M2 phenotype on pigmentation. Intriguingly, our results showed that the conditional medium of E2-treated M2 macrophages significantly increased melanin production in both NHEMs and organ-cultured human skin samples. Consistent with the in vitro results, E2 supplement prominently reversed ovariectomy-decreased epidermal pigmentation in mice, which was dependent on macrophages and linked with M2 polarization. These findings potently demonstrate that estrogen could stimulate melanogenesis via promoting M2 skewing. Supporting our results, M2 macrophages were found to enhance the melanin synthesis-related gene expression in cocultured melanocytes differentiated from human-induced pluripotent stem cells [16]. Although estrogen had been shown to directly upregulate melanin synthesis in human melanocytes in vitro [33], the melanocytes from some donors did not respond to estrogen, even at a high dose [5]. Estrogen was also revealed to indirectly stimulate hyperpigmentation through increasing neighboring cells around melanocytes to produce pro-melanogenic factors. For instance, estradiol could enhance the production of granulocyte-macrophage colony-stimulating factor in keratinocytes via the PKC and ERK pathways [34]; E2 via estrogen receptor-α increased VEGF expression, whereas via GPER upregulated enhanced endothelin-1 content in endothelial cells [35]. Recently, growing evidence points toward a significant immune-driven component in melasma [7]. In addition to its essential role in sexual development and reproduction in females, estrogen has long been recognized to exert crucial roles in regulating immune cell function, particularly in macrophage polarization [36]. In the skin, macrophages are abundant and have a critical function in aging, wound healing, inflammatory diseases, and tumor via polarization [37]. Thus, we consider that pro-melanogenesis by estrogen-induced M2 skewing is a novel function of skin macrophages and might play a crucial role in melasma. Nevertheless, mast cells had been reported as the predominant immune cell type associated with melasma and generally considered to play an important role in melasma development [38,39,40]. In agreement, we also found that resting mast cells were the largest population of infiltrated immune cells in melasma lesions and significantly increased in lesions compared with those in perilesional skin through the transcriptomic analysis. Whether M2 polarization is regulated by mast cells in melasma needs further study. We hypothesize that estrogen-induced M2 macrophages may possibly interact with mast cells and other immune cells, which may subsequently contribute to melasma.

To further corroborate the pro-melanogenic effect of estrogen-induced M2 polarization, the underlying mechanism was next investigated. Regardless of M2 subgroups, M2 macrophages are typically anti-inflammatory in nature and promote apoptotic cell clearance, cell proliferation, tissue repair, and angiogenesis via producing large amounts of cytokines, especially TGF-β, VEGF, and IL-10 [9]. TGF-β1 and VEGF have been reported to affect melanin production in melanocytes [14,15,16]. Yet, IL-10 has not been found to directly affect melanogenesis. We found that E2 could stimulate M2 macrophages to produce and secrete VEGF and TGF-β, but not stem cell factor and endothelion-1. Also, the dermal VEGF level significantly increased in melasma lesions, whereas there was no differentially expressed TGF-β between lesions and perilesional skin. Notably, both VEGF knockdown and the antibody against VEGF165/121 profoundly attenuated the melanogenesis increased by E2-treated M2 macrophages in vitro or in ex vivo skin. Particularly, E2 administration effectively reversed ovariectomy-decreased VEGF level and melanin content of mouse tail skin, which were markedly blocked by the macrophage depletion. According to previous studies, VEGF-A activates p38 and ERK1/2 MAPKs following binding to its receptors [27], which are crucial in melanogenesis [41]. We further found that p38 MAPK and ERK1/2 pathways were activated in melanocytes by the conditional medium of E2-treated M2 macrophages, which were significantly blocked by VEGF antagonism. Importantly, inhibition of either p38 MAPK or ERK1/2 signaling substantially ameliorated the melanogenesis induced by E2-skewed M2 macrophages. These data strongly suggest that estrogen may promote melanogenesis mainly through increasing VEGF production and secretion in M2 macrophages and subsequently activating p38 MAPK and ERK1/2 pathways in melanocytes. However, either VEGF knockdown or antibody could partially ameliorate pro-melanogenic effect of E2-treated M2 macrophages, implying that VEGF, together with other cytokines, contributes to E2-induced melanogenesis.

The upregulated VEGF expression has earlier been noticed in melasma lesions compared to perilesional normal skin and contributes to melasma [42]. Given that VEGF is a potent pro-angiogenic factor, it had been revealed to stimulate hyperpigmentation in melasma via increasing vascularity [43]. Furthermore, VEGF was found to facilitate vascular endothelial cells to produce endothelion-1 [7], which subsequently activated MITF phosphorylation and increased tyrosinase level [7]. Accordingly, vessel-targeting therapies have been proven to be effective in treating melasma. Recently, studies showed that VEGF could directly increase melanin production [15,16]. Nonetheless, the pathogenesis of enhanced VEGF remains largely unknown in melasma. Prior studies initially showed that ultraviolet radiation activated keratinocytes to express VEGF and believed that keratinocytes were the primary source of the increased VEGF in melasma lesions [44]. Also, Kim et al. found that VEGF expression in keratinocytes was significantly enhanced in melasma lesions, whereas the dermal VEGF level was comparable between perilesional and lesional skin [42]. In contrast, several kinds of dermal cells have been identified to contribute to increased VEGF in melasma, including mast cells, senescent fibroblasts, and vascular endothelial cells [4]. We presented that the dermal VEGF expression was significantly increased and mainly colocalized with M2 macrophages in melasma lesions, indicating that estrogen-induced M2 phenotype might possibly serve as an important source of upregulated VEGF in melasma. More confirmatory studies are required to solve this difference. Additionally, investigations with scRNA-seq of human melasma tissues are needed to potentially elucidate the relative contribution of M2 macrophages versus other cells to increased VEGF expression in melasma.

While the results of our study are promising, there are several limitations. First, the number of human melasma specimens was relatively limited, and our transcriptomic data were acquired from public GEO database. Second, in vitro experiments only used THP-1 cells rather than primary human macrophages and did not investigate the effect of the direct blockade of estrogen receptor or VEGFR2 on estrogen-induced macrophage polarization or melanogenesis. Third, clodronate liposomes were used to deplete macrophages in mice, which are not specific for M2 macrophages. Finally, we explored whether estrogen affected the pigmentation of the mouse tail skin via inducing macrophage polarization. However, the mouse tail skin does not fully represent human facial melasma.

In conclusion, this study shows that estrogen-induced M2-like macrophage polarization and VEGF-mediated signaling may contribute to melanogenesis in melasma. Our findings provide the novel mechanism of estrogen-promoted melanogenesis in melasma. Targeting M2 skewing may be a new approach to treat melasma.

## 4. Materials and Methods

### 4.1. Reagents

Phorbol 12-myristate 13-acetate (PMA, P8139), β-estradiol (E2, E2758), and lipopolysaccharides (LPS, L4391) were purchased from Sigma-Aldrich (St. Louis, MO, USA). Recombinant human IL-4 (CX03) and IFN-γ (300-02) were from NovoProtein (Suzhou, China) and PeproTech (Cranbury, NJ, USA), respectively. p38 MAPK inhibitor SB203580 (HY-10256), MAPK/extracellular signal-regulated kinase 1/2 inhibitor U0126 (HY-12031A), and STAT6 phosphorylation inhibitor AS1517499 (HY-100614) were from MedChemExpress (Monmouth Junction, NJ, USA). VEGF165/121 monoclonal antibody (69025-1-IG) was from Proteintech (Wuhan, China). Clodronate liposomes and control liposomes (F70101C-NC) were from FormuMax (Sunnyvale, CA, USA).

PMA, E2, SB203580, and U0126 were dissolved in dimethyl sulfoxide (DMSO) (Biosharp, Hefei, China) at the corresponding concentration. LPS, IL-4, and IFN-γ were dissolved in phosphate-buffered saline (PBS) (Biosharp, Hefei, China) at the corresponding concentration. During cellular experiments, the solution was diluted with the corresponding cell culture medium.

### 4.2. Ethics Statement

For human skin biopsy and skin sample collection, the written informed consent was signed by all donors. Parents, on behalf of their children enrolled, also signed an informed consent form. The experimental programs were approved by the Medical Ethical Committee of Third Affiliated Hospital of Sun Yat-Sen University, Guangzhou, China ([2021] 02–035–01 (31 May 2021), RG2024-092-02 (15 July 2024), RG2024-092-03 (27 April 2025)).

### 4.3. Cell Culture

THP-1 cells purchased from Procell (CL-0233, Wuhan, China) were cultured in RPMI-1640 medium supplemented with 10% fetal bovine serum, 1% penicillin-streptomycin, and 0.05 mM β-mercaptoethanol. They were seeded at a density of 1 × 10^5^ cells/cm^2^ in six-well plates. Following the incubation of 100 ng/mL PMA for 24 h, THP-1 cells were differentiated into M0 macrophages. M0 cells were washed with PBS and then incubated with 10^−7^ M E2 for another 24 h. For M1 polarization, untreated or E2-treated M0 cells were incubated with 100 ng/mL LPS and 20 ng/mL IFN-γ for 72 h, whereas M2 macrophages were generated using 20 ng/mL IL-4. After polarization, culture medium was removed, and cells were washed twice with PBS. Subsequently, RPMI-1640 complete medium was replenished. Then, 24 h later, supernatants from the macrophages were collected for subsequent treatment of melanocytes.

Normal human epidermal melanocytes (NHEMs) at passages 2–5 were maintained in M254 medium supplemented with 1% human melanocyte growth supplement and 1% penicillin-streptomycin. To investigate macrophage–melanocyte interactions, melanocytes were cultured in a 1:1 mixture of the conditioned medium from macrophages and melanocyte culture medium.

For PMA, E2, and inhibitor treatments, control group received the same volume of DMSO as the treated groups, and the final DMSO concentration was kept below 0.1% in all experimental groups.

### 4.4. Melasma Skin Biopsy Collection

Six patients with melasma (mean age 45.33 ± 5.05 years) were enrolled. Skin tissues (4 mm) were excised using a sterilized loop drill from lesional and perilesional normal skin (usually within 1 cm from the lesion margin). All subjects were Fitzpatrick skin type III or IV. The age and sex of each patient were shown in Table S1. These skin samples were subsequently processed for HE staining, multiplex immunofluorescence, and immunohistochemical staining.

### 4.5. Ex Vivo Skin Organ Culture

Normal human skin samples obtained during surgery were cut into approximately 0.5 cm × 0.5 cm sections and placed in a six-well plate. They were maintained in DMEM supplemented with 10% FBS and 1% penicillin/streptomycin or cultured with 50% medium from macrophages. The medium was refreshed every 2 days. After the 5-day incubation, skin samples were harvested for analysis. The experiments were repeated independently three times.

### 4.6. Animal Experiments

Animal experiments were performed in accordance with the 3R principles and received ethical approval from the Animal Ethics Committee of South China Agricultural University, Guangzhou, China (approval code 2024h047, approved on 11 November 2024). Female 8-week-old C57BL/6J mice were maintained on a regular 12 h light/dark cycle with free access to standard mouse chow and water. Mice were randomly divided into five groups. Each group had six mice. They were subjected to a sham operation or ovariectomy under anesthesia using 2′,2′,2′-tribromoethanol (250 mg/kg). Slow-release E2 pellets were implanted subcutaneously in the back area (0.18 mg, 60-day release pellets, Shinnobio, Suzhou, China) immediately after ovariectomy. A starting dose of 200 μL clodronate liposomes or control liposomes (F70101C, Formumax, Sunnyvale, CA, USA) was intraperitoneally injected 3 days after ovariectomy, followed by 150 μL for each subsequent injection twice a week for 6 weeks. All the group allocation, treatment assessment, image analysis, and histological quantification were performed in a blinded manner.

### 4.7. RNA-Seq Data Access and Immune Infiltration Analysis

We searched the Gene Expression Omnibus (GEO) database with the criteria of published human melasma skin transcriptome data and obtained a set of transcriptome data (GSE72140) comparing melasma lesions with perilesional tissue (n = 11) [45]. These data were first preprocessed to exclude missing and duplicate values. Then, samples were classified into the lesional and perilesional groups, resulting in a gene expression matrix suitable for downstream analyses. Afterward, immune infiltration analysis was performed on the acquired data using the CIBERSORT R package (v0.1.0) with the LM22 dataset, which quantified and compared the infiltration scores of 22 immune cell subsets between melasma lesions and perilesional skin.

### 4.8. MEGCNA Analysis

To identify the gene co-expression network and hub genes in melasma, we used MEGCNA. Compared to Weighted Gene Co-expression Network Analysis (WGCNA), MEGCNA reduces the false-positive rate and computational complexity, significantly enhancing performance. To improve computational accuracy and reduce false-positive findings, we first extracted the top 5000 genes with the highest average expression levels from the gene expression matrix and then conducted MEGCNA analysis. The threshold of a significant *p*-value of both modules and hub genes was set at 0.05.

### 4.9. VEGF Knockdown in THP-1 Cells with Lentivirus Transduction

Lentiviruses for VEGF knockdown (sh-VEGF) and negative controls (sh-NC) were produced and verified by GenePharma company (Shanghai, China). Upon reaching 60% confluence, THP-1 cells were infected with lentivirus vectors at 40 MOI for 12 h and then incubated with fresh medium for additional 48 h. Following that, cells were treated with 1 μg/mL puromycin to select stably transfected cells for subsequent experiments. The transduction efficiency was verified 4 days after infection by examining VEGF expression with qPCR and Western blotting. The experiments were repeated independently three times.

### 4.10. RNA Extraction and Real-Time Quantitative PCR (qPCR)

Real-time PCR analysis was performed to examine gene expression in human skin, mouse skin, macrophages, and melanocytes. Total RNA was extracted using RNA Extraction Kit (B0132, Haigene, Harbin, China) according to the manufacturer’s instructions and quantified spectrophotometrically. A total of 1 μg of total RNA was reverse-transcribed into cDNA using a PrimeScript RT reagent kit (RR037, Takara Bio, Shiga, Japan) at 37 °C for 15 min and 85 °C for 5 s. RT-qPCR was performed with the PrimeScript ^TM^ RT reagent Kit (RR037, Takara Bio, Shiga, Japan) and TB Green Premix Ex Taq II (RR820, Takara Bio, Shiga, Japan) according to the following conditions: initial activation at 95 °C for 30 s, followed by 40 cycles of 95 °C for 5 s and 60 °C for 30 s. Primers for PCR reaction are shown in Table S2. Gene expression was quantified using the comparative Ct method. Relative expression levels of target genes were calculated using 2^−ΔΔCt^ as the fold increase over controls. The experiments were repeated independently three times.

### 4.11. Western Blot Analysis

Cells were lysed using a whole cell lysis assay kit (KGB5303, Keygen, Nanjing, China). Then, 20 μg total protein was resolved using 10% SDS-PAGE (Invitrogen, Carlsbad, CA, USA) and transferred to a polyvinylidene difluoride membrane (MilliporeSigma, Burlington, MA, USA). After being blocked by 5% BSA in tris-buffered saline with 0.1% Tween, membranes were incubated with primary antibodies against phosphorylated p38 MAPK (4511T, Cell Signaling Technology, Danvers, MA, USA), p38 MAPK (8690T, Cell Signaling Technology, Danvers, MA, USA), phosphorylated ERK1/2 (4390T, Cell Signaling Technology, Danvers, MA, USA), ERK1/2 (4695T, Cell Signaling Technology, Danvers, MA, USA), MITF (A1255, ABclonal, Wuhan, China), TYR (A1254, ABclonal, Wuhan, China), VEGFA (AF5131, Affinity, Cincinnati, OH, USA), VEGFR2 (AF6281, Cincinnati, OH, USA), and GAPDH (2118T, Cell Signaling Technology, Danvers, MA, USA), respectively, at 4 °C overnight. After subsequent incubation with appropriate secondary antibodies, protein bands were detected with enhanced chemiluminescence reagents. Blots were photographed and visualized with FluorChem M system (ProteinSimple, San Jose, CA, USA). The intensity of bands was quantified with ImageJ software (v1.54g). For quantification, we quantified all the bands that were only located within the standard molecular weight range of the detected protein. All values were normalized to the corresponding GAPDH. The experiments were repeated independently three times.

### 4.12. Flow Cytometry

Cells were collected from six-well plates and washed three times with PBS. Then, cells were resuspended in flow cytometry staining buffer (00-4222-57, Thermo Fisher, Waltham, MA, USA) and incubated with Fc receptor blocking antibody (14-9161-71, Thermo Fisher) at 20 °C for 20 min. Subsequently, cells were added with PE anti-human CD86 (12-0869-41, Thermo Fisher, Waltham, MA, USA) or PE-Cy7 anti-human CD206 (25-2069-41, Thermo Fisher, Waltham, MA, USA) and incubated at 4 °C in the dark for 60 min. Following two additional washes, cells were analyzed by flow cytometry, and the expression of cell surface molecules was measured with BD FACSCanto (BD Bio-sciences, San Jose, CA, USA) according to manufacturer’s instructions. The experiments were repeated independently three times.

### 4.13. Immunohistochemistry Staining

For skin samples from patients and mice, the paraffin-embedded tissue slices were deparaffinized, rehydrated, and subjected to antigen retrieval. After blocking with normal goat serum at room temperature, slices were incubated with primary anti-VEGF (CY5096, Abways, Shanghai, China), anti-F4/80 (70076, Cell Signaling Technology, Danvers, MA, USA), anti-CD86 (19589, Cell Signaling Technology, Danvers, MA, USA), anti-CD206 (24595, Cell Signaling Technology, Danvers, MA, USA), and anti-TGF-β (21898-1-AP, Proteintech, Wuhan, China) antibodies, respectively, overnight under 4 °C at 1:100 dilution. Sections were then incubated with appropriate secondary antibodies. After staining with DAB solution, sections were counterstained with hematoxylin.

### 4.14. Immunofluorescence Staining

To examine the phenotype of infiltrated macrophages and VEGF colocalization with macrophages in melasma lesions or mice skin, multiplex immunofluorescence staining was performed to examine CD68, CD86, VEGF, CD206, and F4/80. The collected skin specimens were stored in formalin solution and then embedded in paraffin. The slices were incubated with anti-CD68 (ab201340, Abcam, Cambridge, MA, USA)/anti-CD86 (91882s, Cell Signaling Technology, Danvers, MA, USA)/anti-CD206 (24595, Cell Signaling Technology, Danvers, MA, USA), or anti-CD68/anti-CD206/anti-VEGF (CY5096, Abways, Shanghai, China), or anti-F4/80 (70076, Cell Signaling Technology, Danvers, MA, USA)/anti-CD206 (24595, Danvers, MA, USA)/anti-CD86 (19589, Cell Signaling Technology, Danvers, MA, USA) antibodies at 1:400 dilution, respectively, and kept overnight at 4 °C. Then, the slices were incubated with secondary antibodies, and cell nuclei were counterstained with 4′,6-diamidino-2-phenylindole (DAPI). All images were scanned using 3DHIESTECH digital scanner (Budapest, Hungary) and analyzed using CaseViewer software (v2.4) (Budapest, Hungary). The experiments were repeated independently three times.

For immunofluorescence staining of the in vitro cultured cells, cells were fixed with 4% paraformaldehyde in coverslips and then incubated with CD206 or CD86 antibody at 1:500 dilution (DF4149, Affinity, Cincinnati, OH, USA) overnight under 4 °C. Afterward, cells were incubated with the secondary antibody, and nuclei were counterstained with DAPI. The images were acquired using a fluorescence microscope (DM4000B, LEICA, Wetzlar, Germany). The experiments were repeated independently three times.

### 4.15. Fontana-Masson Staining

Fontana–Masson staining was used to measure melanin content in human and mouse skin sections. The procedure followed manufacturer-recommended protocols (G2032, Solarbio, Beijing, China). Briefly, paraffin-embedded tissue sections were deparaffinized and rehydrated. Sections were subsequently immersed in 10% silver nitrate solution at 60 °C water bath in the dark for 30 min. Then, skin sections were transferred to 0.2% gold chloride, fixed in 5% sodium thiosulfate, and finally counterstained with nuclear fast red solution. The images were captured in the bright field microscope (Nikon, Tokyo, Japan) and quantified by the percentage of pigmented area per epidermal area (PA/EA) using ImageJ software.

### 4.16. Melanin Content and Tyrosinase Activity Assay

Melanin content and tyrosinase activity were detected in melanocytes. Normal human epidermal melanocytes were lysed with radioimmunoprecipitation assay buffer (9806, Cell Signaling Technology, Danvers, MA, USA). The cellular lysates were centrifuged at 4 °C at 12,000 rpm for 15 min. For melanin content assay, cell pellets were dissolved in 1 N sodium hydroxide at 60 °C for 2 h. Following this, 100 µL of the solution was added to 96-well plates. OD values were measured at 490 nm with a microplate reader (Bio-Rad, Hercules, CA, USA) and compared with a standard curve of synthetic melanin (Sigma-Aldrich, St. Louis, MO, USA). The experiments were repeated independently three times.

Tyrosinase activity was measured by incubating 100 mL supernatant of melanocyte lysate with equal volume of 2 mM Levodopa (PHR1271, Sigma-Aldrich, St. Louis, MO, USA) in 0.1 M phosphate buffer (pH 6.8) at 37 °C for 90 min. After incubation, the absorbance was measured at 490 nm with a microplate reader (Bio-Rad, Hercules, CA, USA). The melanin content and tyrosinase activity were normalized to the protein content of each sample. The experiments were repeated independently three times.

### 4.17. Enzyme-Linked Immunosorbent Assay (ELISA)

To study the mechanism of enhanced melanogenesis by E2-induced M2 macrophages, vascular endothelial growth factor (VEGF), stem cell factor (SCF), endothelin-1 (ET-1), and transforming growth factor-β (TGF-β) were detected in supernatants with ELISA. Levels were detected using ELISA kits (MEIMIAN, Yancheng, Jiangsu, China) according to the manufacturer’s instructions. The absorbance at 450 nm was measured with a microplate reader and compared with standard curves. The concentration of VEGF, SCF, ET-1, and TGF-β was normalized to the protein content of each sample. The experiments were repeated independently three times.

### 4.18. Statistical Analysis

All data were analyzed using the Windows version of GraphPad Prism 9.0. The data are presented as means ± standard deviation (SD). Student’s paired or unpaired *t*-test was used to examine the statistical significance of two groups, while statistical analyses among three or more groups were performed with one-way ANOVA, followed by a Bonferroni’s multiple comparison test. Paired Wilcoxon rank-sum test was performed to analyze gene expression differences of transcriptome data between melasma lesions and perilesional skin. Correlations were assessed with Pearson correlation coefficient analysis. A *p*-value less than 0.05 was considered statistically significant, * *p* < 0.05, ** *p* < 0.01, *** *p* < 0.001, and **** *p* < 0.0001.

## Data Availability

The data presented in this study are available on request from the corresponding author due to ethical reasons.

## Supplementary Materials

The following supporting information can be downloaded at: https://www.mdpi.com/article/10.3390/ijms27136044/s1.
