# Supplementary material for: Estrogen Promotes Melanogenesis Through Facilitating M2 Macrophage Skewing in Melasma
Source: Int J Mol Sci. 2026 Jul 6;27(13):6044. doi: 10.3390/ijms27136044 (PMC13361750; doi:10.3390/ijms27136044)
Supplement: Supplementary file 1 [file ijms-27-06044-s001.zip › ijms-4374819-supplementary.pdf]

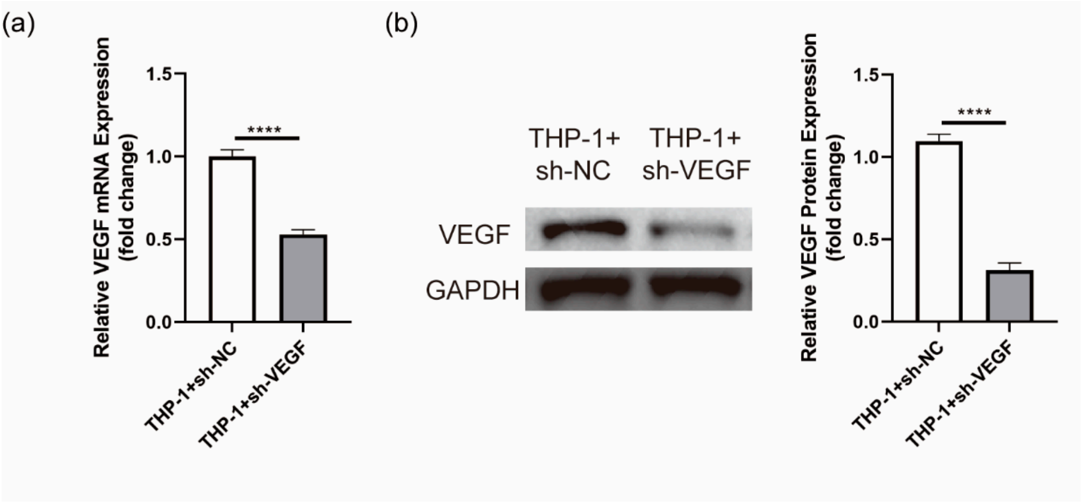

Supplementary Figure S1. The efficiency of VEGF knockdown in THP-1 cells with lentivirus transduction. (**a-b**) mRNA (**a**) and protein (**b**) expression of VEGF in THP-1 cells transduced with VEGF-knockdown lentiviral vectors (sh-VEGF) or empty vectors (sh-NC). THP-1 cells were infected with lentiviral vectors at 40 MOI for 12 h. After puromycin selection, stably transfected cells were cultured and harvested 4 days post infection for analysis of mRNA and protein levels of VEGF. Data were presented as means ± SD from three independent experiments. ****P* <0.0001 by Student' *t*- test.

Supplementary Table S1. The sex and age of melasma patients.

Patients	Sex	Age
1	Female	52
2	Female	50
3	Female	45
4	Female	44
5	Female	43
6	Female	38

Supplementary Table S2. The sequence of primers for qPCR.

Gene	Sequence
Human-GAPDH-F	GGAGCGAGATCCCTCCAAAAT
Human-GAPDH-R	GGCTGTTGTCATACTTCTCATGG
Human-IL-10-F	GCTGTCATCGATTTCTTCCC
Human-IL-10-R	TCAAACTCACTCATGGCTTTGT
Human-TNF-α-F	CCTCTCTCTAATCAGCCCTCTG
Human-TNF-α-R	GAGGACCTGGGAGTAGATGAG
Human-iNOS-F	TTCAGTATCACAACCTCAGCAAG
Human-iNOS-R	TGGACCTGCAAGTTAAAATCCC
Human-VEGF-F	GAGCCTTGCCTTGCTGCTCTA
Human-VEGF-R	CACCAGGGTCTCGATTGGATG
Human-ARG1-F	GTGGAAACTTGCATGGACAAC
Human-ARG1-R	AATCCTGGCACATCGGGAATC
Human-MITF-F	CTCACAGCGTGTATTTTTCCCA
Human-MITF-R	ACTTTCGGATATAGTCCACGGAT
Human-TYR-F	GCAAAGCATACCATCAGCTCA
Human-TYR-R	GCAGTGCATCCATTGACACAT
Mouse-Gapdh-F	AGGTCGGTGTGAACGGATTTG
Mouse-Gapdh-R	TGTAGACCATGTAGTTGAGGTCA
Mouse-VEGF-F	CACGACAGAAGGAGAGCAGAAG
Mouse-VEGF-R	CTCAATCGGACGGCAGTAGC
Mouse-iNOS-F	GTTCTCAGCCCAACAATACAAGA
Mouse-iNOS-R	GTGGACGGGTCGATGTCAC
Mouse-Arg1-F	CTCCAAGCCAAAGTCCTTAGAG
Mouse-Arg1-R	GGAGCTGTCATTAGGGACATCA
